# Supplementary material for: Association between oral microbiome and seven types of cancers in East Asian population: a two-sample Mendelian randomization analysis
Source: Front Mol Biosci. 2023 Nov 21;10:1327893. doi: 10.3389/fmolb.2023.1327893 (PMC10702768; doi:10.3389/fmolb.2023.1327893)
Supplement: Supplementary file 1 [file DataSheet1.ZIP › Supplementary Materials/MR plots of saliva/saliva═╝/Pancreatic cancer/pheno.1421_to_pancreatic cancer_forest.pdf]

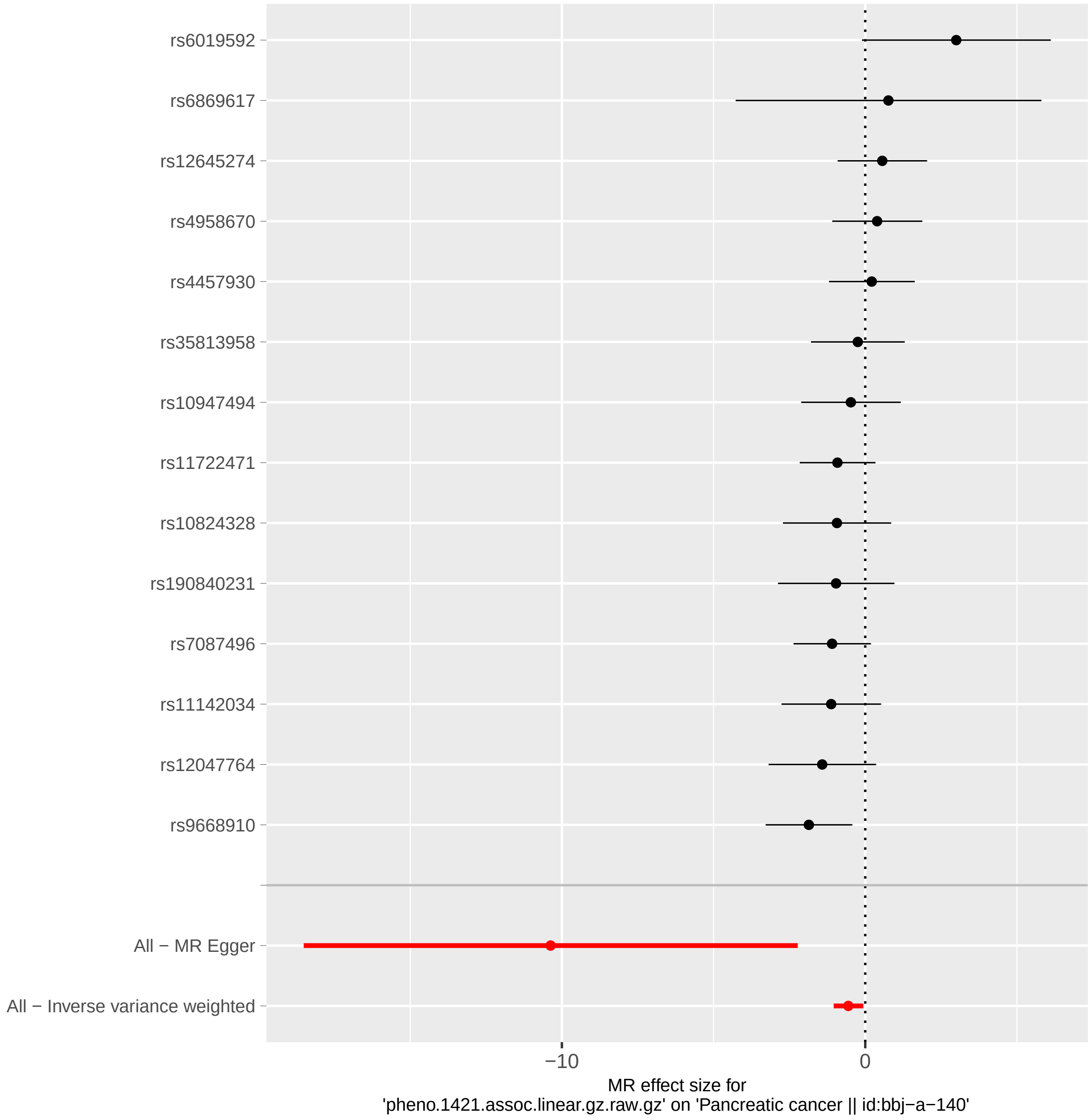
rs6019592
rs6869617
rs12645274
rs4958670
rs4457930
rs35813958
rs10947494
rs11722471
rs10824328
rs190840231
rs7087496
rs11142034
rs12047764
rs9668910
All − MR Egger
All − Inverse variance weighted
−10
0
MR effect size for
'pheno.1421.assoc.linear.gz.raw.gz' on 'Pancreatic cancer || id:bbj−a−140'
